# Deficits in tapping accuracy and variability in tremor patients

**DOI:** 10.1186/s12984-019-0528-6

**Published:** 2019-05-07

**Authors:** Frauke Luft, Sarvi Sharifi, Winfred Mugge, Alfred C. Schouten, Lo J. Bour, Anne-Fleur van Rootselaar, Peter H. Veltink, Tijtske Heida

**Affiliations:** 1 Department of Biomedical Signals and Systems, University of Twente, Enschede, The Netherlands; 2 Department of Neurology, Amsterdam Neuroscience, Amsterdam UMC, University of Amsterdam, Amsterdam, The Netherlands; 3 Department of Mechanical, Maritime and Materials Engineering, Delft University of Technology, Delft, The Netherlands

**Keywords:** Parkinson’s disease, Essential tremor, Tapping accuracy, Tapping variability, Kinetic tremor, Auditory cue

## Abstract

**Background:**

The basal ganglia and cerebellum are brain structures involved in movement initiation, execution and termination. They are thought to be involved in the tremor generation and movement deficits in Parkinson’s disease (PD) and essential tremor (ET). Especially in PD, maintaining cyclic movement, such as walking or tapping can be significantly disturbed. Providing external cues improves timing of these movements in PD but its effect on ET has not yet been studied in depth. The aim of this study is to evaluate the usefulness of a bimanual tapping task as a tool during clinical decision making.

**Method:**

Hand movements and tremor was recorded using accelerometers and EMG (m. extensor carpi ulnaris) from PD and ET patients and healthy controls during a bimanual tapping task as a way to distinguish PD from ET. All subjects performed the tapping task at two different frequencies, 2 Hz and 4 Hz, with and without the presence of auditory cues.

**Results:**

No significant intra-group differences were found in the patient groups. Acceleration data revealed significantly less accurate tapping and more variable tapping in PD than in ET and healthy controls. ET subjects tapped less accurate and with a greater variability than healthy controls during the 4 Hz tapping task. Most interestingly the tapping accuracy improved in PD patients when kinetic tremor was recorded with EMG during the task.

**Conclusion:**

Providing ET and PD patients with an external cue results in different tapping performances between patient groups and healthy controls. Furthermore, the findings suggest that kinetic tremor in PD enables patients to perform the task with a greater accuracy. So far this has not been shown in other studies.

## Background

Movement disorders can affect all phases of movement, i.e. the preparation, timing, execution and termination. Two of the most common movement disorders are Parkinson’s disease (PD) and essential tremor (ET) [1, 2]. PD is considered a disease of the basal ganglia, and for ET, literature suggests pathological changes in the cerebellum [3]. However, the classifications of the two disorders have been revised in recent years. Classic PD is diagnosed based on bradykinesia and at least one of the following symptoms: rigidity, or resting tremor [4, 5]. ET is clinically defined as a disorder, presenting with a bilateral postural or/and kinetic tremor [6–8]. However, it has been shown that ET is a rather heterogeneous disorder making the diagnosis difficult when patients present with a resting tremor as well as a postural or kinetic tremor [7–9]. On the other hand, PD patients may express a postural tremor, similar to the one seen in ET and no resting tremor [10]. Furthermore, rigidity and postural instability are two symptoms usually associated with PD, but are also common in elderly [11], complicating the diagnosis of elderly PD (most people with idiopathic PD develop symptoms at 50 years of age or older [12]) and late-onset ET patients (late disease onset at age 46 and older [13]). Since tremor is a common disease characteristic, many studies have attempted to distinguish PD and ET based on quantitative characteristics of tremor such as tremor amplitude and frequency [14]. However, the ranges of these tremor characteristics show significant overlap despite the pathological differences and differences in classic clinical presentation [15]. Therefore, the rate of misdiagnosis in ET is up to 37% with the most common true diagnosis either PD (15%) or dystonic tremor [16, 17].

The Unified Parkinson’s Disease Rating scale (MDS-UPDRS), commonly used to rate the severity of PD, includes various finger and hand movement tasks. The parameters of interest are the speed, amplitude and regularity of these movements. These parameters have also been studied quantitatively in several studies [18, 19]. PD patients often suffer from a difficulty to initiate movements, including standing up or starting to walk, and to continue rhythmic cyclic motion, including walking and finger tapping [19, 20]. Other studies have shown that external cues enhance PD patients’ ability to start and continue movements [20–22]. Several studies have looked into patients’ ability to perform finger tapping tasks when cueing was applied [19, 21]. During these experiments, PD patients, in particular tremor dominant PD patients, tended to hasten movements when finger tapping tasks at lower frequencies (< 3 Hz) had to be performed. At higher frequencies (4–5 Hz) patients tapped slower than the cue frequency [21]. Furthermore, studies using a synchronization-continuation task also showed that PD patients hastened movements at lower frequencies and that this deficit was greater in PD patients with freezing of gait episodes [23]. A pilot study by Espay et al. [18] found that patients with PD tapped with a smaller amplitude and reduced speed during an index finger to thumb tapping task compared to healthy controls (HC).

Fewer studies have been conducted on ET patients and rhythmic cyclic movements. Farkas et al. [24] found an impairment of rhythm generation and an increased variability of hand movement during externally cued movements compared to HC. Furthermore, Avanzino et al. [25] found that 1 Hz-rTMS over the ipsilateral lateral cerebellum affected the performance during a finger movement task in patients with ET by reducing touch duration values and normalizing the inter touch interval values.

The ability to perform a rhythmic cyclic movement has been evaluated for both patient groups in comparison with healthy controls, but not in comparison with each other or other neurological disorders. Additionally, such a task is not included in any rating scale (MDS-UPDRS and Tremor rating scale (TETRAS)), even though differences between patients and healthy controls are known and adding such a task might be useful during clinical decision making or analyzing disease progression. Furthermore, the occurrence of kinetic tremor during such a movement has not been evaluated, even though, kinetic tremor has been reported in both disorders and is the primary symptom in ET. Therefore, the aim of this study is to evaluate and compare the performance of ET and PD patients in performing a bimanual synchronization-continuation tapping task to determine the usefulness of such a task as a tool during clinical decision making. For this purpose we evaluated movement characteristics during rhythmic cyclic movements at different frequencies. Furthermore, we evaluated the effect of kinetic tremor on these movement characteristics. An additional group of HC was included to evaluate overall deficits in both groups compared to HC. We expected to find greater deficits in timing ability in the PD group and more frequent occurrence of kinetic tremor in the ET group compared to the other two groups.

## Methods

### Participants

Eleven PD patients (3 Female (F); Age: 66 ± 11) (mean ± std. (years)), seventeen ET patients (4 F; Age 58 ± 17) and nineteen HC (9 F; Age: 59 ± 15) participated in the study. HC subjects were matched in age as good as possible to both groups (see Table 1). No significant differences in age between the three groups was found (PD – ET: *p* = 0.29; PD – HC: *p* = 0.28; ET – HC: *p* = 0.98). Gender was not considered to have any influence on the results. For the patient groups an overview of the tremor frequencies, recorded with accelerometers, of resting, postural and kinetic tremor are given in Table 2. All subjects were right-handed according to the Edinburgh Handedness Inventory [26] and patients were willing to stop medication according to an individual scheme prior to the experiment. Furthermore, the PD patients were diagnosed with Parkinson’s disease according to the UK Brain Bank criteria for Parkinson’s disease [27] and showed neither major fluctuations in symptoms nor suffered from severe dyskinesia. Additionally, all PD patients presented with a resting tremor during the intake. ET patients had essential tremor according to the criteria defined by the Tremor Investigation Group [28], and had a positive family history of ET. HC had no record of neurological or other diseases affecting the motor system. The study was conducted in accordance with the Declaration of Helsinki and was approved by the Medical Ethical Committee of the Academic Medical Center, Amsterdam, The Netherlands. All subjects signed written informed consent prior to the experiment.

**Table 1:** Overview of included subjects

Subject	Gender	Age	Duration (Years)	Medication
PD 1	M	69	5	Levodopa, Trihexyphenidyl
PD 2	M	67	4	Propranolol, Rasagilin
*PD 3	F	81	5	Trihexyphenidyl
*PD 4	M	62	2	Levodopa, Metoprolol succinat
PD 5	M	71	2	Levodopa
PD 6	F	43	3	–
PD 7	M	68	8	Levodopa, Rasagilin, perindopril, omeprazole
PD 8	M	64	15	–
PD 9	M	67	11	Levodopa, Benserazid
*PD 10	F	56	9	Levodopa, Carbidopa, Amandatin
PD 11	M	64	7	–
ET 1	M	50	50	–
ET 2	F	81	21	–
* ET 3	M	54	4	Propranolol
ET 4	M	85	Childhood	Propranolol
* ET 5	M	65	Childhood	–
ET 6	F	51	Childhood	–
* ET 7	M	65	45	Propranolol
ET 8	F	23	Childhood	–
ET 9	M	49	9	Propranolol
ET 10	M	54	Childhood	–
ET 11	M	70	Childhood	–
ET 12	M	64	Childhood	–
ET 13	M	55	Childhood	–
* ET 14	M	72	12	Propranolol
ET 15	M	27	Childhood	–
ET 16	F	81	Childhood	–
ET 17	M	45	Childhood	–

*PD* = Parkinson’s Disease, *ET* = Essential Tremor, *M* = Male and *F* = Female, *R* = Right hand, *L* = Left hand. The asterisk * marks the subjects that were either excluded (Essential Tremor and Healthy Controls) due to technical failure or false execution of the task or not able to perform the 4 Hz tapping task (Parkinson’s disease)

**Table 2:** Overview tremor frequencies

Subject	Rest (Hz) (R/L)	Posture (Hz) (R/L)	Tapping (Hz) (R/L)
PD 1	5/5	6/5	6/6
PD 2	5/5	7/8	−/6
*PD 3	6/4	5/7	−/5
*PD 4	6/6	7/7	7/7
PD 5	9/8	6/6	−/6
PD 6	8/8	9/8	−/−
PD 7	4/5	5/6	6/6
PD 8	6/6	6/7	−/−
PD 9	7/6	6/7	5/−
*PD 10	6/6	5/5	−/−
PD 11	6/7	6/8	−/−
ET 1	6/7	6/6	−/−
ET 2	5/5	7/6	6/5
* ET 3	–	–	–
ET 4	8/6	5/6	−/−
* ET 5	–	–	–
ET 6	7/6	7/7	7/7
* ET 7	–	–	–
ET 8	8/7	7/8	−/8
ET 9	7/7	5/6	7/−
ET 10	6/6	6/7	6/6
ET 11	5/9	7/7	−/−
ET 12	7/4	7/7	9/−
ET 13	8/8	8/8	8/7
* ET 14	–	–	–
ET 15	6/6	5/7	−/7
ET 16	5/5	5/10	6/6
ET 17	7/7	6/7	−/−

*PD* = Parkinson’s Disease, *ET* = Essential Tremor, *R* = Right hand, *L* = Left hand. The tremor frequencies are given for each individual patient. For the postural tremor patients had both arms outstretched in front of them at shoulder level

### Experimental setup

Subjects were seated on a bed, with head and back supported for a sitting posture. They performed a bimanual, in-phase, tapping task at two predefined cue frequencies, 2 and 4 Hz (equivalent to an inter-tap interval of 500 ms and 250 ms, respectively). The forearm and proximal part of the wrist joint were supported against gravity by the bed and tapping movements were made by wrist flexion. Subjects had to continuously tap with both hands on the bed next to their legs for three minutes at each frequency. The three minutes were split into blocks of thirty seconds, with alternately the auditory cueing switched on and off. Instructions were given verbally prior to the experiment and all subjects were able to perform the task without further practice. During cued movement, subjects were able to hear a metronome beat via computer speakers. Prior to the experiment subjects were asked whether they were able to hear the sound clearly. The three-minute tapping task was performed at both cue frequencies.

Hand movement was recorded with 3D accelerometers (TMSi, Oldenzaal, The Netherlands) attached to the back of each hand, approximately in the middle of the third metacarpal bone. Sensor size is 13x10x5 mm and sensor weight is 2 g.

Muscle activity was recorded from the m. extensor carpi ulnaris of both arms using surface electromyography (EMG) electrodes. All signals were connected to a physiological amplifier system (Porti, TMSi, Oldenzaal, The Netherlands) to synchronize the data streams. Data acquisition was done with a sampling frequency of 2048 Hz using a customized program written in LabVIEW (National Instruments, Woerden, The Netherlands).

In this study EMG and accelerometers were used to record movement and tremor as they may provide complementary information.

### Data pre-processing

First the norm of the accelerometer vectors was calculated, giving one acceleration signal for each hand. The acceleration signal for each hand was filtered off-line (non-causal, 0.25–20 Hz bandpass, 4th order Butterworth). Afterwards the data was split into cued and non-cued movement, the first and last 2 s of each block were removed to avoid transition effects, giving 3 blocks of 26 s for each condition. This was done for the 2 Hz and for the 4 Hz tapping task. EMG data was filtered off-line (non-causal, 20–400 Hz, 4th order Butterworth). Then the absolute value of the Hilbert transform was used as the envelope of the EMG signal for further analysis.

### Data analysis

Acceleration and EMG data were split into 3 s epochs with 50% overlap. The power spectral density (PSD) of each epoch was estimated using a periodogram with a rectangular window to maximize frequency resolution. For each epoch the tapping frequency was calculated as the highest peak in a range +/− 1 Hz of the cue frequency, resulting in the spectrum between 1 and 3 Hz and between 3 and 5 Hz for the 2 Hz and 4 Hz tapping tasks, respectively. These narrow frequency bands were chosen after visual inspection of all epochs and subjects. To test the timing ability and the consistency of tapping we use two measures that are retrieved from the acceleration data, the tapping accuracy (TA) and tapping variability (TV).

The tapping accuracy (TA):

1 $$ {\mathrm{TA}}_{\mathrm{Subject}}=\frac{\sum \limits_{\mathrm{n}=1}^{\mathrm{N}}\left({\mathrm{f}}_{\mathrm{cue}}-{\mathrm{f}}_{\mathrm{tap},\kern0.5em \mathrm{n}}\right)}{\mathrm{N}} $$

With *f*_*cue*_ the predefined cue frequency, *f*_*tap*,  *n*_ the tap frequency per epoch, *n*, the epoch number and *N* the total number of epochs. A TA of 0 resembles perfect synchronization with the cue frequency.

The tapping variability (TV):

2 $$ {\mathrm{TV}}_{\mathrm{Subject}}=\frac{\sum \limits_{\mathrm{n}=1}^{\mathrm{N}}\left|\left({\mathrm{f}}_{\mathrm{mean}}-{\mathrm{f}}_{\mathrm{tap},\kern0.5em \mathrm{n}}\right)\right|}{\mathrm{N}} $$

With *f*_*mean*_ the mean tapping frequency during cued and non-cued movement, *f*_*tap*, *n*_ the tapping frequency in epoch n, and N the total number of epoch. TV = 0 indicates a perfectly constant tapping frequency. The variability was calculated in addition to the accuracy to see whether subjects tapped a constant rhythm independent of the predefined cue frequency. TA and TV were also calculated from the EMG data. This was done to determine whether the measurement signals provided complementary information about movement characteristics.

The EMG data was analyzed with respect to the occurrence of kinetic tremor. For this purpose a tremor frequency band was chosen between 5 and 14 Hz [29]. The presence of kinetic tremor was determined quantitatively using a straight forward threshold method and by visual inspection of the PSD of the EMG data of each subject. An epoch was classified as kinetic tremor if the ratio of the average power in the tremor band (5–14 Hz) and the average power in the movement band (1–5 Hz) exceeded a threshold level of 0.8, according to Eq. 3:

3 $$ \mathrm{Kinetic}\ \mathrm{Tremor}:\frac{\sum \limits_{\mathrm{f}=5}^{\mathrm{f}<14}\mathrm{Pxx}\left(\mathrm{f}\right)/{\mathrm{N}}_{\mathrm{TF}}}{\sum \limits_{\mathrm{f}=1}^{\mathrm{f}<5}\mathrm{Pxx}\left(\mathrm{f}\right)/{\mathrm{N}}_{\mathrm{MF}}}\ge 0.8 $$

**Fig. 1:**
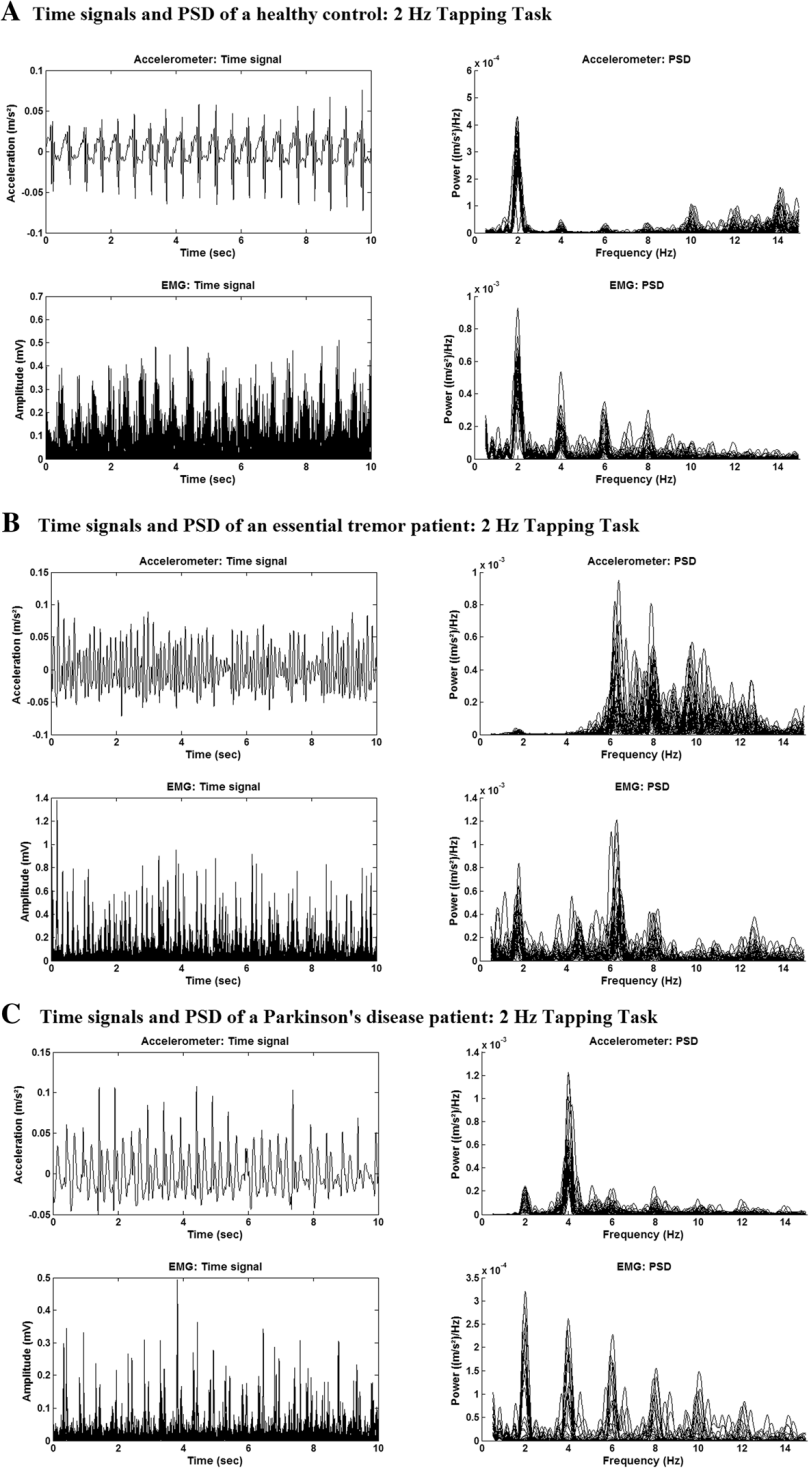
Example of the time signal and corresponding power spectral density. Time signal (10 s) and power spectral density (PSD) (of all epochs) of the EMG (top) and accelerometer (bottom) data. Time signals are presented in the left figure and the PSD in the right Fig. **a**) Data are of a representative healthy subject. **b**) Data are of an ET patient with high power kinetic tremor at 6 Hz. **c**) Data are of a PD patient with high power kinetic tremor at 4 Hz

With Pxx(f) the power at each frequency, f,, *N*_*TF*_ the number of samples in the tremor frequency band and *N*_*MF*_ the number of samples in the movement band (1–5 Hz). The threshold of 0.8 was chosen based on the data of the HC assuming that they did not show kinetic tremor. The mean ratio of the power in the tremor band and the movement band was 0.41 in the HC, 0.86 in the ET and 1.01 in the PD group. The threshold was chosen as approximately twice the mean ratio of the HC group. Kinetic tremor was only determined using the EMG data, because the occurrence of high-power higher harmonics of the movement in the accelerometer data (see Fig. 1) made the use of a straight forward threshold condition difficult.

All parameters were calculated for cued and non-cued movement during the 2 and 4 Hz tapping task and for the left and the right hand.

### Statistical analysis

To determine differences in accuracy and variability during cued and non-cued movements (movement condition) with the left and right hand (hand) a multiway analysis of variance (ANOVA) is used. A *p*-value smaller than 0.05 was considered significant. The following intra-group differences were analyzed for the 2 Hz and the 4 Hz tapping task:

- Difference in TA and TV between the left hand cued, right hand cued, left hand non-cued and right and non-cued movement.
- Difference in TA and TV: main factor hand
- Difference in TA and TV: main factor movement condition

The inter-task difference between the 2 Hz and the 4 Hz tapping task (left and right hand separately) was determined.

In addition, the following inter-group differences were analysed for the 2 Hz and the 4 Hz tapping task:

- Difference in TA and TV: main factor group.
- Difference in TA and TV: main factor group and interaction factor movement condition
- Difference in TA and TV: main factor group and interaction factors movement condition and hand.

## Results

After visual inspection of the data 2 HC and 1 ET patient were excluded due to technical failure of one or more accelerometers or EMG electrodes. 3 ET patients performed the tapping task by only using their index finger and not their whole hand. These subjects were also excluded from further analysis to prevent errors due to differences in task execution. 3 PD patients (marked in Table 1) were not able to perform the 4 Hz tapping task due to fatigue or other reasons. Eventually, 17 HC, 12 ET subjects, 11 PD subjects (2 Hz tapping task) and 8 PD subjects (4 Hz tapping task) were included in the analysis.

In Fig. 1, data of a HC (A), an ET (B) and PD (C) patient is given. At the top, 10 s of the pre-processed EMG data and the corresponding PSD plots are shown. The figure below shows the accelerometer data and corresponding PSD plots for the same time frame of 10 s. In the PSD plot (EMG and accelerometer) of the ET and PD patient two main peaks are visible. One is located around the cue frequency of 2 Hz, the tapping frequency, and the other around 6 Hz, the kinetic tremor frequency. In the accelerometer data also a peak at 4 Hz is visible. This peak is most likely a higher harmonic of the tapping frequency.

### Tapping accuracy

**Fig. 2:**
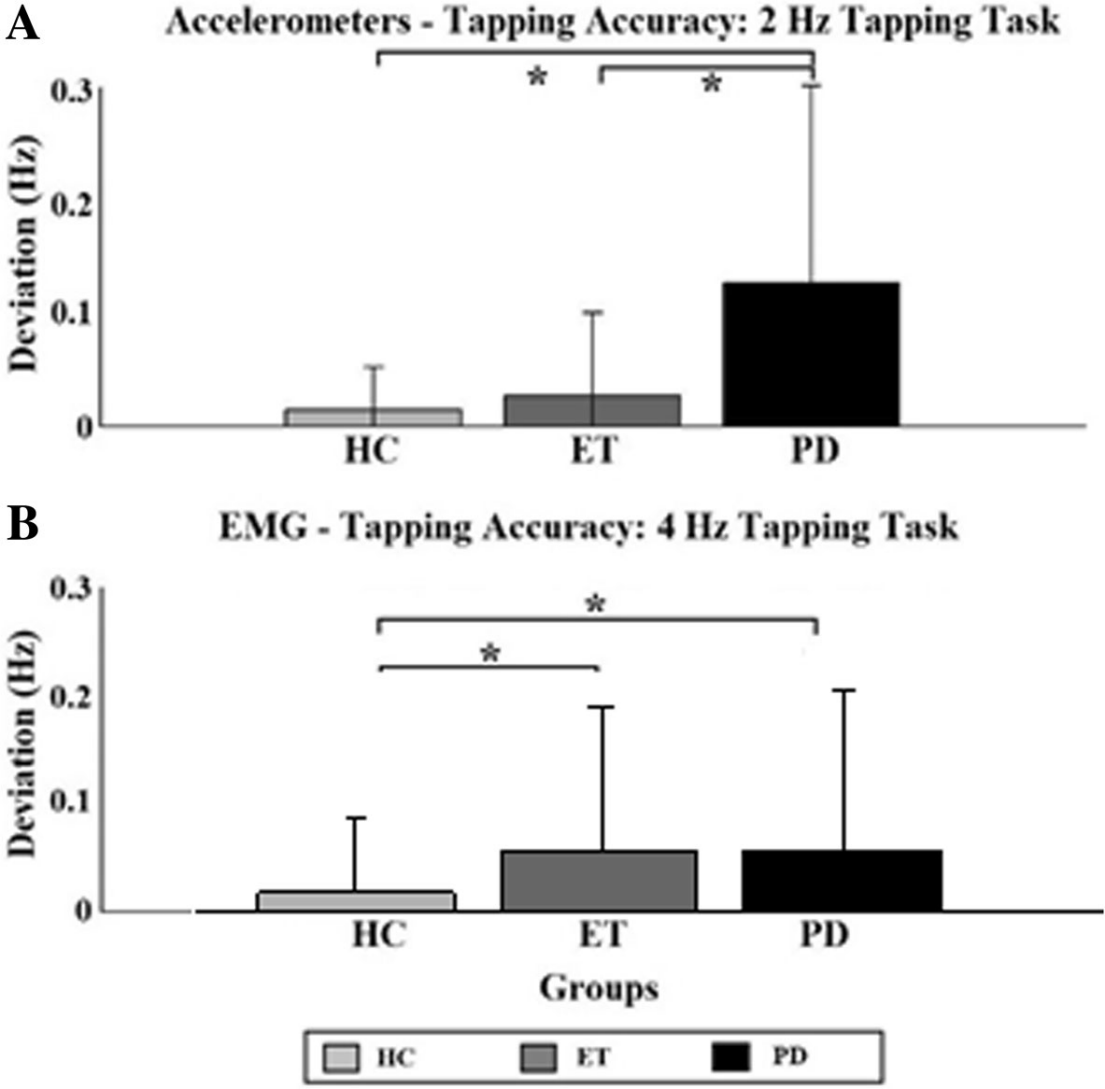
Tapping accuracy. Tapping accuracy determined with the accelerometers for the 2 Hz (**a**) and 4 Hz (**b**) tapping task. Significant differences between individual task/hand combination are indicated by asterisks. HC = Healthy controls (light grey); ET = Essential Tremor (dark grey); PD = Parkinson’s Disease (black)

No significant intra-group differences in TA were found. Therefore, the inter-group analysis was only done for the main factor “group” and interaction factor “movement condition”. In Fig. 2 the results that showed significant inter-group differences are displayed: the results of the accelerometer data for the 2 Hz (A) and the results of the EMG data for the 4 Hz tapping task (B). The bar plots give the absolute value of the deviation from the cue frequency of each group and the error bars indicate the standard deviations of the groups.

Inter-group differences for the 2 Hz tapping task were found in the accelerometer data. The PD group tapped significantly less accurate than the other two groups (PD: 0.12 and 0.08; ET: 0.02 and 0.08; HC: 0.01 and 0.02, for the 2 Hz and 4 Hz tapping task respectively) (indicated by asterisks). Considering only cued movement, they tapped less accurate than the HC group.

EMG data revealed significant differences during the 4 Hz tapping task between the patient groups and the HC group (indicated by asterisks). The HC group tapped with greater accuracy than the two patient groups. Both patient groups tapped slower (mean ± std.: 3.99 ± 0.19 Hz and 3.98 ± 0.16 Hz for PD and ET, respectively) than the predefined cue frequency of 4 Hz, whereas the HC group either tapped accurately at the cue frequency or tended to tap faster than that (mean: 4.02 ± 0.10 Hz).

### Tapping variability

**Fig. 3:**
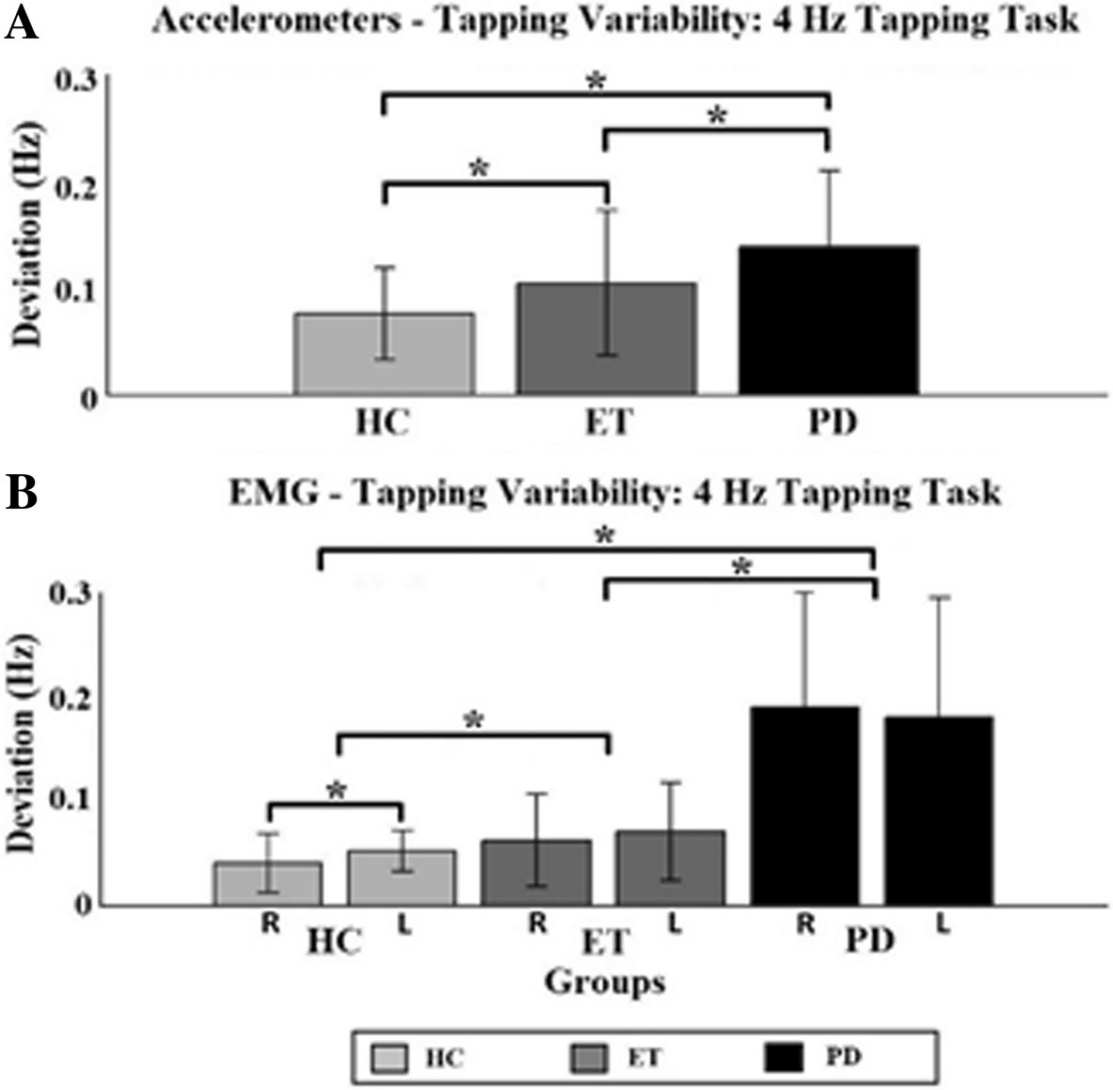
Tapping variability. Tapping variability determined with the accelerometers for the 4 Hz (**a**) and EMG data of the 4 Hz (**b**) tapping task. Inter-group differences between individual task/hand combination are indicated by two asterisks. HC = Healthy controls (light grey); ET = Essential Tremor (dark grey); PD = Parkinson’s Disease (black); R = Right hand; L = Left hand

In Fig. 3 the significant results concerning the tapping variability are displayed. The only significant intra-group difference found was in the HC group between the right (dominant) and left hand during the 4 Hz tapping task in the EMG data (see bottom part of Fig. 3). Significant differences were only found during the 4 Hz tapping task. The accelerometer data found significant inter-group (main factor “group”) differences during the 4 Hz task between all three groups. PD patients tapped with a significantly higher variability than the other two groups. ET patients tapped with a greater variability than the HC group (indicated by asterisks).

EMG data showed significant differences during the 4 Hz tapping task as well. PD group tapped with greater variability than the other two groups (indicated by asterisks). And ET with a greater variability than the HC group.

### Kinetic tremor

**Fig. 4:**
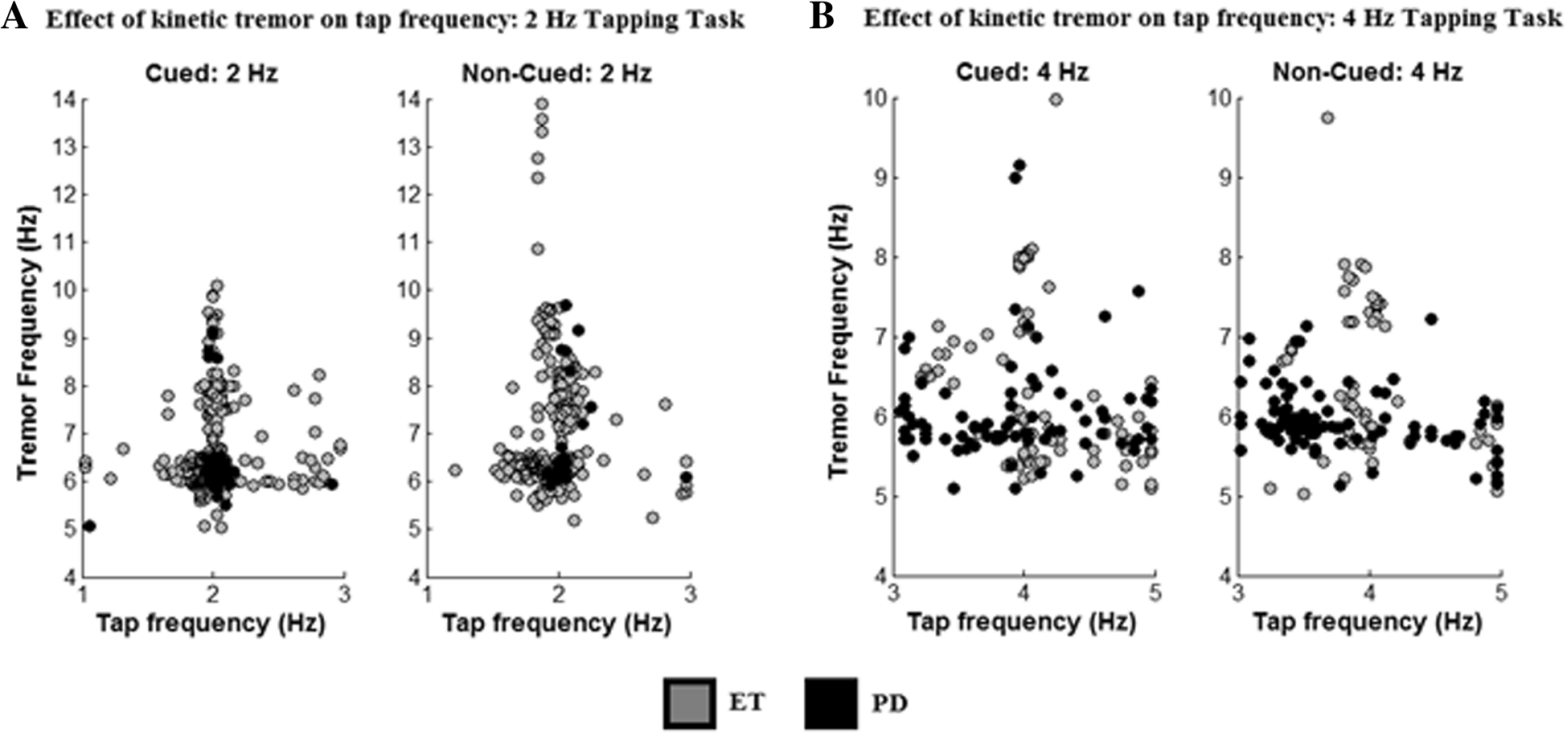
Effect of kinetic tremor on the tapping frequency. Tapping frequency vs. kinetic tremor frequency, determined from EMG data, during the 2 Hz (**a**) and 4 Hz (**b**) tapping task. In black the results of the PD group are given and in grey the results of the ET group

Kinetic tremor was detected electrophysiologically in nine of the thirteen ET patients using our tremor detection method. In three subjects tremor occurred during both tasks, at both hands and during both tapping conditions. In six subjects (46%) kinetic tremor was detected during the cued movement at 2 Hz. During non-cued movement five ET patients (38%) showed a kinetic tremor at the right and six at the left hand. During the 4 Hz tapping task four subjects (31%) showed tremor at the right hand, during the cued and non-cued movement. Five subjects presented with tremor at their left hand. In eight of the eleven PD patients tremor was detected electrophysiologically during at least two of the eight hand-condition-task combinations. Three subjects (27%) presented with a tremor in the right hand during the 2 Hz tapping task. At the left hand one subject (9%) showed a tremor during cued movement and three subjects during non-cued movement. During the 4 Hz tapping task (only eight subjects) three subjects (38%) experienced tremor during cued movement. Non-cued movement caused a kinetic tremor in one subject (13%) at the right and in two subjects (25%) at the left hand. In Fig. 4 it is shown that during the 2 Hz tapping task (A), the PD subject experiencing tremor tapped more accurate and with a smaller variability compared to ET subjects with kinetic tremor.

During the 4 Hz tapping task (B) both groups tend to tap with a high variability when tremor occurs. A trend is visible indicating that PD patients tend to tap slower than the predefined frequency during non-cued movement, whereas the presence of tremor does not seem to have an effect on the variability in the ET group.

## Discussion

We hypothesized that kinetic tremor would occur more frequent in ET patients compared to the other two groups. However, kinetic tremor occurred in both patient groups to the same extent. The second hypothesis that PD patients suffer greater deficits in TA and TV compared to the other two groups is accepted. Differences exist between PD and ET patients in performing rhythmic cyclic movements under cued and non-cued conditions. First, the mean tapping frequency during the 4 Hz tapping task is significantly lower in the PD group than the ET group. Second, acceleration data revealed significantly less accurate tapping in the PD group than the ET group during the 2 Hz tapping task. Third, acceleration and EMG data revealed a greater tapping variability in the PD group during the 4 Hz tapping task compared to the ET group. Fourth, occurrence of kinetic tremor had a different effect on TA and TV in the ET group compared to the PD group.

### Tapping accuracy and variability

Overall, the PD group tapped less accurate than the other two groups. This was the case for the cued and non-cued movement during the 2 Hz tapping task and consistent with the findings of Freeman at. al [21]. These findings are consistent with the hypothesis that the basal ganglia play mainly a role in the internal timing of voluntary repetitive movements, and that this dysfunction can to some extent be compensated for by external cues. The PD group tapped at a lower frequency than the cue frequency during the 4 Hz tapping task. This is in accordance with the findings of Freeman et al. [14] and could be due to bradykinesia affecting these patients. In general, the ET subjects also tapped slower than the cue frequency of 4 Hz. Therefore, it can be concluded that ET as well as PD patients experience a timing problem during rhythmic, cyclic movements. Other studies have shown that patients with cerebellar dysfunction or lesion show a larger variability in rhythmic movement as was seen in the ET group [30]. The cerebellum is involved in the timing of movement. It acts as an internal timing system and this system is disturbed in ET patients [31]. Furthermore, differences in activity in the cerebellum exist during a finger tapping task in ET compared to healthy controls as has been shown by Buijink et al. [27]; in this study ET patients showed decreased activation in widespread areas of the cerebellar cortical regions compared to healthy controls. On the other hand, altered activity in the cerebellum is thought to be the cause of kinetic tremor. Therefore, these widespread changes might lead to a timing problem in the cerebellum in ET patients, a timing problem between executing voluntary movement (tapping) and causing involuntary movement such as tremor. In PD external cueing is thought to take over the function of the basal ganglia as an internal clock by activating the cerebellum as a compensatory pathway. However, the activation of the cerebellum due to the cue also causes an increased kinetic tremor in some patients. Helmich et al. [32] introduced the dimmer-switch model for parkinsonian resting tremor in which it is suggested that the basal ganglia activates tremor (switch), and the cerebello-thalamo-cortical circuit modules tremor amplitude (dimmer). This concept might also be applicable for kinetic tremor in case of cued movement. As a compensatory mechanism the PD group can tap more accurately by bypassing the basal ganglia, while this activation of the cerebellar circuit also affects tremor. In the ET group the tapping frequency seemed unaffected by the occurrence of tremor. This could explain the differences in TA between PD and ET during the 2 Hz task. Cueing affects the activity in the cerebellum, which in case of ET may have an adverse effect on motor output due to the dysfunction of the cerebellum. In a future study we hope to further explore these theories with the use of imaging data. The differences seen between the 2 Hz and the 4 Hz tapping task is most likely simply a consequence of the tempo subjects had to follow. At 2 Hz subjects are able to react to each single cue as a separate event, whereas at 4 Hz a pattern generator is most likely activated.

### Kinetic tremor

Kinetic tremor is commonly found in ET, but also in some PD patients [33] which can cause confusion between the two, especially in early stages of the disease when other symptoms of PD are not as distinctive as in the later stages. Our results support the findings of others that kinetic tremor is also found in PD patients even though it is less common than in the ET group. However, our findings show that the occurrence of tremor influences the TA and TV of the patient groups differently. Where it seemed to have no effect on the ET group it influenced the PD group during the 2 Hz task. This is rather intriguing because the group analysis shows that overall the PD group taps less accurate than the ET group, even though, Fig. 4 indicates that PD subjects with kinetic tremor tap more accurate than subjects with ET showing tremor. To our knowledge these finding have not been reported in other studies and suggests that different tremor mechanisms exist in these two disorders.

Four ET patients did not suffer from kinetic tremor during the repetitive movements. Louis et al. [34] showed that the occurrence of resting tremor in ET depends on the study setting. Our results suggest that the same could be true for kinetic tremor in ET. Furthermore, the ET and PD patients presenting with tremor during the tapping task had asymmetric and symmetric tremor presentation (Table 2). This indicates that tremor symmetry is not a good indicator for differentiation between PD and ET.

### Study limitations

Due to malfunctioning of the basal ganglia in PD patients the external cue is thought to replace the missing internal cue [29]. However, no differences were found between the two tapping conditions, neither at 2 Hz nor at 4 Hz. It could be that the paradigm used in this study was not suitable to detect such differences. Thirty seconds of cued movement was immediately followed by thirty seconds of non-cued movement. It is possible that cueing has a wash-out effect which has a positive influence on the TA during the non-cued movement task.

Another study [20] concerning the effect of cueing during physiotherapy has found indications for such an effect. Therefore, it is suggested to slightly adapt the paradigm of this study to yield a larger intra-group effect by including a resting period between cued and non-cued movement. This might also result in a larger inter-group difference as not only the continuation of cyclic movement would be included in the analysis but also the effect of initiating a cyclic movement without an external cue. To determine differences between the dominant and non-dominant hand in subjects, tapping with the left and right hand should be separated into two different tasks. Furthermore, the current setup and choice of sensors is based on a clinical setup where the way of executing the movement is known. Induced kinetic tremor was expected in the extensor muscles and could therefore be measured by choosing these muscles to record from. This allowed us to measure kinetic tremor from the EMG data.

Movement was recorded using accelerometers placed on both hands and EMG recordings from the m. extensor carpi radialis of the left and right forearm. Accelerometers record the start of movement of an extremity whereas EMG records the activation of a muscle that could result in movement but not necessarily has to. Results were in no case contradicting each other. Differences could be caused by the activation of additional or other muscles, not measured here with surface EMG, influencing the net movement of the hand. Therefore, in addition to the extensor muscle the flexor muscle might also be of interest. It could show if the flexion movement of the hand, necessary for tapping, is caused by activation of the flexor muscle or rather by the relaxation of the extensor muscle. The results of this study show that the measurement techniques can provide supplementary information as also shown in other disciplines, such as prosthetics [35]. Especially in case of kinetic tremor, EMG might be able to detect it earlier and locate its origin more accurate than accelerometers. In some cases kinetic tremor was only visible in the EMG data and not in the accelerometer data. This indicates that the occurrence of a tremor in a muscle does not always have to cause visible tremor in an extremity. It might be though that this tremor in the muscle leads to fatigue of the muscle due to this overactivity [36] and therefore to less or no movement. Another possibility is that the occurrence of tremor inhibits the movement by inhibiting movement selection whenever the power in the tremor frequency band is larger than in the movement band [37].

The results suggest that this method might be useful in a classifier model for movement disorders. In this model Step one would be to separate patients from healthy controls. And the second would be to separate patient groups from one another using the mean tapping frequency during a 4 Hz tapping task and the effect of kinetic tremor on TA and TV during a 2 Hz tapping task.

## Conclusion

This study showed that with a straightforward experimental setup consisting of two accelerometers and two surface EMG electrodes differences between PD and ET patients can be revealed during a bimanual tapping task. Furthermore, differences between both patient groups and HC were found. Especially the finding that the occurrence of kinetic tremor did not affect tapping accuracy of PD patients while it decreased tapping accuracy in ET could be used in a classifier model to support clinical decision making during the diagnostic process of PD and ET.
